# Radioimmunotherapy for mantle cell lymphoma: 5-year follow-up of 90 patients from the international RIT registry

**DOI:** 10.1007/s00277-020-03956-0

**Published:** 2020-03-03

**Authors:** Karin Hohloch, Christine Windemuth-Kieselbach, Pier Luigi Zinzani, Roberto Cacchione, Wojciech Jurczak, C. Suh, Lorenz Trümper, Christian W. Scholz

**Affiliations:** 1 Department of Hematology and Oncology, Kantonsspital Graubünden, Chur, Switzerland; 2 Alcedis GmbH, Independent CRO, Giessen, Germany; 3 Department of Hematology and Oncology, Georg August University, Goettingen, Germany; 4 Institute of Hematology “Seràgnoli”, Università di Bologna, Bologna, Italy; 5 Médica e Investigaciones Clínical, “Norberto Quirno”, CEMIC, Centro de Educación, Buenos Aires, Argentina; 6 Department of Haematology, Jagiellonian University, Cracow, Poland; 7 Department of Hematology and Oncology, Vivantes Klinikum Am Urban, Berlin, Germany; 8 Asan Medical Center, Department of Internal Medicine, Division of Oncology, University of Ulsan College of Medicine, Seoul, South Korea

**Keywords:** Radioimmunotherapy, Mantle cell lymphoma, Outcome

## Abstract

To assess the efficacy of radioimmunotherapy (RIT) with ^90^yttrium-ibrutinib-tiuxetan (90Y-IT) in mantle cell lymphoma, data from 90 patients registered in the RIT Network with a median follow-up (FU) of 5.5 years after RIT were evaluated. 90Y-IT was given as first-line therapy in 45 (50%) and for relapse in 45 (50%) patients. Most patients received 90Y-IT as consolidation after chemoimmunotherapy in first line (98%) and in relapse (53%). As a first-line treatment, 30 patients (pts.) (67%) achieved CR, 10 pts. (22%) PR%. and 1 pt. (2%) PD, and for 4 pts. (9%), no response data was available. At relapse, CR was achieved in 17 pts. (38%), PR in 6 pts. (13%), SD in 2 pts. (4%), and 6 pts. (13%) had PD, while the response was not documented for 14 pts. (31%). After a median FU of 5.5 years, median PFS for all patients was 2.11 (95% CI, 1.03–2.32) years, and median OS was 4.05 (95% CI, 2.79–7.21) years. Eleven pts. (12.2%) developed second malignancy. In conclusion, this is the largest report of MCL pts. treated with 90Y-IT to date. 90Y-IT was most often used as consolidation after first- and second-line chemotherapy and may improve the results achieved using chemoimmunotherapy alone. However, the results are less encouraging compared to treatment with small molecules such as ibrutinib.

## Introduction

Mantle cell lymphoma (MCL) is a radiosensitive disease. Current guidelines recommend involved field radiotherapy (IF-RT) in patients with limited non-bulky stages I and II tumours, preferably as consolidation after a shortened conventional chemotherapy [1]. However, the majority of MCL patients are diagnosed during stage III or IV and are treated with a chemoimmunotherapy, including the anti-CD20 antibody rituximab alongside a chemotherapy, which is chosen according to the performance status of the patient. Younger, more fit patients are treated with induction chemotherapy followed by high-dose chemotherapy (HCT) and autologous stem cell transplantation (ASCT), while less fit individuals receive treatments such as R-CHOP (rituximab, cyclophosphamide, doxorubicin, vincristine, prednisone), R-B (rituximab bendamustine), or R-CAP (rituximab, bortezomib, cyclophosphamide, doxorubicin, prednisone). Recently, addition of rituximab maintenance therapy has led to a survival benefit for patients treated with R-CHOP alone or in combination with HCT and ASCT [2, 3]. Radioimmunotherapy (RIT) with the anti-CD20 antibody ibritumomab connected with tiutexan to the radionucleotide ^90^yttrium combines immuno- and radiotherapy treatment modalities. ^90^yttrium-ibritumomab-tiuxetan (90Y-IT) (Zevalin®) is approved for the treatment of follicular lymphoma, i.e. as consolidation after first-line chemo(immuno)therapy or at relapse. 90Y-IT can be applied in an outpatient setting. Side effects are well managed, most frequently occurring is cytopenia between weeks 6 and 9 after treatment, and infections are not common as well as need for transfusions.

Currently, 90Y-IT is not licensed for the treatment of mantle cell lymphoma (MCL). There are published data from only a few prospective small clinical trials in first [4, 5] or later therapy lines [6]. Furthermore, data of long term follow in view of progression-free survival (PFS), overall survival (OS) and secondary malignancies are missing. In 2006, the RIT network (RIT-NT) was founded as a web-based registry using an electronic data capture system for patients B cell non-Hodgkin’s lymphoma (NHL) treated with radioimmunotherapy. We show here the results of 90 patients with MCL registered in the RIT-NT treated with 90Y-IT with a medium follow-up of 5.5 years.

## Patients and methods

Between December 2006 and November 2009, the RIT-NT was active in 14 countries. The RIT-NT evolved from pre-existing national RIT registries in Spain, Austria, Switzerland and Germany. A web-based electronic data capture (EDC) system was utilized for documentation of the patients. The main dataset includes indication, age, haematologic toxicity, lymphoma subtype and clinical course. For the evaluation of specific projects, such as the here presented, datasets from national registries were brought together with the international databases. National approval of the ethics committees (EC) was received by the respective national RIT registry chairs. Written informed consent of patients was mandatory. Data was collected and stored de-identified in the database of the registry. An institutional IRB vote was provided by the EC of the University of Göttingen.

The registry is maintained by a professional clinical research organization (CRO; Alcedis GmbH, Giessen, Germany). Further information regarding the EDC system has been published recently [7]. For the analysis presented here, all initially participating centres with documented mantle cell lymphoma (MCL) patients in the RIT-NT were contacted via mail to take part in the extended follow-up for MCL. For documentation, the web-based electronic data capturing (EDC) system was used.

## Results

Data from 1105 lymphoma patients treated between December 2006 and November 2009 with radioimmunotherapy were documented in the RIT-NT. Out of these 1105 patients, 135 suffered from MCL. Six countries participated with 90 patients in the elongated follow-up analysis (Poland *n* = 53, Germany *n* = 24, South Korea *n* = 4, Italy n = 4, Switzerland *n* = 3 and Argentina n = 2), while centres from 8 countries that initially participated in the registry chose not to participate in the report of longer follow-up data for unknown reasons. Follow-up analysis included progression-free survival, overall survival, relapse therapy and second malignancy.

### Patient and disease characteristics

Median follow-up for the 90 MCL patients treated with 90Y-IT was 5.5 years (range 0 to 11.5 years). Median age at diagnosis was 63 years (range 31–78 years), 62% of patients were older than 60 years, and 21% were above 70 years. Seventy percent of patients were male, and only 30% were female (Table 1). Tumour stage was documented only upon initial diagnosis. Using the Ann Arbor classification, 69% of patients had stage IV, 24% stage III, 2% stage II and 3% stage I disease (Table 1). The MIPI score, bulky disease and extranodal involvement information were not on hand, because neither the MIPI score nor the required variables are incorporated in the documentation of the RIT-NT.

**Table 1:** Patient characteristics

No. of patients	90	
Male/female	63	(70%)/27 (30%)
Median age (range)	63	(31–78)
*n* > 60 years	56	(62.2%)
*n* > 70 years	19	(21.1%)
Stage	No. of patients	(%)
	*n*	%
Stage IV	62	68.9%
Stage III	22	24.4%
Stage II	2	2.2%
Stage I	3	3.3%
Missing	1	1.1%
Previous therapies		
Chemo(immuno)therapy	*n*	%
0	4*	(4.4)
1	34*	(37.8)
2	28	(31.1)
3	11	(12.2)
> 4	13	(14.4)
Radiotherapy	*n*	%
0	82	(91.1)
1	7	(7.8)
2	1	(1.1)
Autologous stem cell transplantation		
	*n*	%
	10	(11.11)
Indication for RIT		
Line	n	%
First-line therapy	45	50
Primary therapy (RIT mono)	1	1.1
Consolidation (after first-line th.)	44	48.9
Relapse	45	50
Recurrence	12	13.3
Refractory	3	3.3
Conditioning	2	2.2
Consolidation	24	26.7
No indication	1	1.1
Other	3	3.3

### Previous therapies

In this study, first-line therapy is specified as RIT for primary treatment (monotherapy) or as consolidation after first-line treatment with chemo- or chemoimmunotherapy. Relapse is specified as 90Y-IT given as a monotherapy for relapsed or refractory MCL, as consolidation after chemo(immune)therapy or as section of a conditioning regimen prior to autologous stem cell transplantation. RIT was given as first-line therapy in 45 patients (50%) and for relapse in 45 individuals (50%). In the first-line group, most patients received 90Y-IT as consolidation (*n* = 44, 98%), while only 1 individual (2%) was administered 90Y-IT as a monotherapy as first-line treatment. Likewise, in the relapse group, most patients (*n* = 24, 53%) received RIT as consolidation after immunochemotherapy, 13 pat. received RIT for recurrence, 3 pts. for refractory disease and 2 pts. as part of a conditioning regiment, and for 3 pts., indication for RIT is not documented (Table 2). Of note, 90Y-IT was most often used as first-line therapy (*n* = 45, 50%) and second-line therapy (*n* = 23, 26%), while only 20 patients received RIT as third or higher lines of treatment, and for 2 patients, line of therapy was not documented (Table 2). Radiotherapy prior to RIT was infrequent with 8 (9%) patients receiving radiotherapy before RIT. Most patients had 1 (*n* = 34, 38%) or 2 (*n* = 28, 31%) chemo(immuno)therapies prior to RIT, while 3 chemo(immuno)therapies (*n* = 11, 12%), 4 (*n* = 7, 8%), 5 (n = 3, 3%) 6 (n = 1, 1%) and more than 7 (n = 2, 2%) were less frequent (Table 1). Only 2 pts. had been previously treated with RIT. Of note, in some patients, chemo- and immunotherapy, e.g. COP and rituximab, given as first line, were counted as two therapies. Similarly, omission of one drug from a regimen, e.g. omission of doxorubicin from CHOP resulting in CVP, was counted as two therapies in first line in some cases. Therefore, not 44 but only 34 are listed in Table 1 to have received one chemo(immune)therapy before obtaining 90Y-IT.

**Table 2:** Indication for RIT and line of therapy (*n*)

	Line of therapy					
Indication	First line	Relapse				
	First	All	Second	Third	Fourth	≥ Fifth line
Conditioning		2	1	1		
Consolidation	44	24	15	1	2	6
Primary therapy	1	0				
Recurrence		12	5	2	1	4
Refractory		3	2			1
Other		4	1	1		1
All	45 (50%)	45 (50%)	24 (25.5%)	5 (5.5%)	3 (3.3%)	12 (13.3%)

At any rate, however, 44 patients received RIT as consolidation after first-line immunochemotherapy, and one patient obtained 90Y-IT without any prior treatment. None of the patients had a Bruton’s kinase inhibitor like ibrutinib prior to 90Y-IT.

### Response, duration of response and overall survival

*For all patients, response data is only available after RIT. If RIT was preceded by chemoimmunotherapy, which was frequently the case, response was not typically evaluated directly after induction therapy nor was such a response recorded in the RIT registry. Therefore, information regarding conversion,* e.g. *from PR after induction therapy to CR after RIT, is not available from the RIT registry.* For all patients (*n* = 90), the best response was CR in 47 pts. (52%), PR in 16 pts. (18%), SD in 2 pts. (2%) and PD in 7 pts. (8%). Accurate response data were not reported for 18 pts. (20%). In the first-line group with 45 patients, 30 pts. (67%) achieved complete remission (CR), 10 pts. (22%) achieved partial remission (PR), 1 pt. (2%) experienced progressive disease (PD), not documented was response for 4 pts. (9%). For patients treated in relapse (*n* = 45), the CR rate was 38%, PR was 13%, SD was 4% and 13% had PD, while the response was not documented for 31% (*n* = 14 pts.) (Table 3). With a median follow-up of 5.5 years (range 0–11.5 years), the median PFS for all patients was 2.11 years (95% CI, 1.03–2.32), and median OS was 4.05 years (95% CI, 2.79–7.21). For first-line patients, median PFS was 2.79 (95% CI, 2.14–3.79) years, while it amounted to 0.88 (95% CI, 0.66–1.5) years for relapsed patients.

**Table 3:** Response according to indication

Best response	All patients	First line	Relapse
	*n* (%)	*n* (%)	*n* (%)
CR	47 (52.2)	30 (66.7)	17 (37.8)
PR	16 (17.8)	10 (22.2)	6 (13.3)
SD	2 (2.2)	0	2 (4.4)
PD	7 (7.8)	1 (2.2)	6 (13.3)
Missing	18 (20)	4 (8.9)	14 (31.1)

**Fig. 1:**
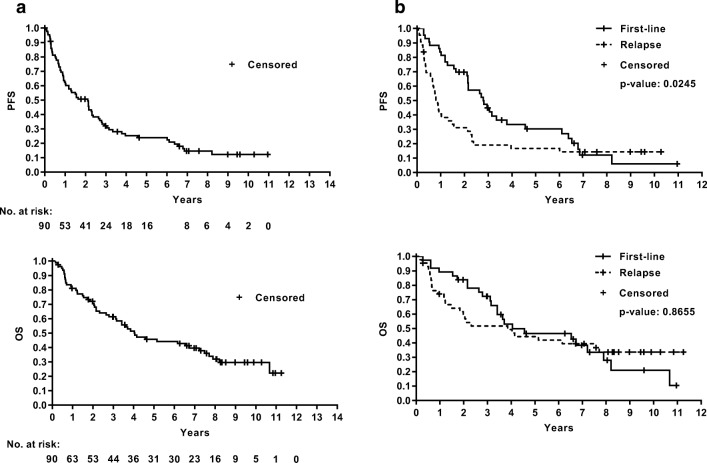
Progression-free survival (PFS, top) and overall survival (OS, bottom) for all patients (**a**) or patients on first-line therapy or with relapse (**b**)

Median OS in the first-line group was 4.05 years (95% CI, 3.15–7.9) and was 3.85 years (95% CI, 1.49–7.71) in the relapse group (Fig. 1).

### Second malignancies

With a median follow-up time of 5.5 years (range 0–11.5 years), in 11 (12%) of the 90 patients, a second malignancy evolved. In nine patients, second malignancy occurred after first-line therapy, and all of these patients had an initial fludarabine-containing regiment (fludarabine, cyclophosphamide [FC], rituximab-FC [R-FC] or R-FC mitoxantrone [R-FCM]). In two patients, second malignancy occurred after 5th and 6th line therapy. Time of onset of secondary malignancies after RIT was not documented in the registry. Of the patients with second malignancies, 6 (55%) suffered from myelodysplastic syndrome (MDS), 2 from prostate cancer, 1 from oesophageal cancer, 1 from NSCLC and 1 from rhabdomyosarcoma.

## Discussion

The RIT registry (RIT-NT) is the largest registry of MCL patients treated with 90Y-IT published to date. Half of the 90 patients reported herein received 90Y-IT as first-line therapy, in most cases as consolidation after chemo- or chemoimmunotherapy. For the remainder, 24 or 26% of patients were given 90Y-IT as second-line treatment, in most cases (15 of 24 pts.) as consolidation after chemo(immuno)therapy.

Overall response rate and CR for patients with first-line therapy were 89% and 67%, respectively. After a median follow-up of 5.5 years, the median PFS and OS for patients treated in first line amounted to 2.79 and 4.05 years, respectively. Toxicity was as expected no unexpected safety signals were detected for employment of 90Y-IT in mantle cell lymphoma. There are few studies employing 90Y-IT as first-line therapy for MCL. In a prospective multicentre trial, 34 patients with MCL were treated as first line with distinct chemo(immuno)therapies (FCM, FC, CHOP or CVP ± R) and received consolidation with 90Y-IT upon achieving a predefined tumour response after 3 to 6 cycles of treatment. 90Y-IT consolidation improved the CR rate in chemosensitive patients from 41 to 91%, and the median PFS and OS amounted to 3.3 and 6.5 years, respectively [5]. In line with these findings, 57 MCL patients were treated in a prospective single-centre trial with 90Y-IT if they had achieved at least stable disease after four cycles of R-CHOP. Herein, the ORR and CR rates were 82% and 52%, respectively, and the median time to treatment failure (TTF) amounted to 34 months [8]. With a longer follow-up median of 9.8 years, median OS for the entire cohort of 56 patients was 7.9 years. During follow-up, one myeloid neoplasia and 6 solid malignancies (2 NSCLC, 1 bladder cancer, 1 ampullary cancer and 2 non-melanoma skin cancers) were observed [4]. These results from 90Y-IT consolidation after shortened chemoimmunotherapy and data from the RIT-NT presented compare well with data from MCL patients treated in clinical trials with six cycles of chemoimmunotherapy with or without rituximab maintenance, i.e. chemoimmunotherapy with R-B (bendamustine), R-CHOP and VR-CAP with or without rituximab maintenance. Here, ORR ranged between 86 and 93%, and the CR rate was between 30 and 53%. Median PFS amounted to 35 months after R-B, ranging from 14 to 22 months after R-CHOP and 24 months after VR-CAP [9, 10]. Treatment of elderly MCP patients with R-CHOP followed by rituximab maintenance induced a 4-year remission duration of approximately 60% [2]. Therefore, an abbreviated chemoimmunotherapy followed by 90Y-IT might be just as effective as six cycles of chemoimmunotherapy with or without rituximab maintenance.

In contrast, a phase 2 trial from the Nordic lymphoma group did not show a benefit from applying 90Y-IT to MCL patients who had only achieved PR after induction chemoimmunotherapy with R-maxiCHOP and high-dose cytarabine and were subsequently treated with HCT and ASCT. Here, the data were compared to a previous trial where patients received the same chemoimmunotherapy, including HCT and ASCT, and did not get 90Y-IT. With respect to PFS and OS, adding 90Y-IT did not improve the results [11], indicating that 90Y-IT may not improve CR rates after intensive induction chemoimmunotherapy.

Regarding relapsed MCL in the RIT-NT, the ORR and CR rates for 90Y-IT as a monotherapy or as consolidation after chemo(immuno)therapy were 51% and 38%, respectively. Median PFS and OS amounted to 0.88 and 3.85 years, respectively. There are little data from prospective clinical trials employing 90Y-IT in patients with relapsed MCL. In the aforementioned multicentre trial [5], 12 patients received 90Y-IT consolidation after responding to 3–6 cycles of second-line chemo(immune)therapy. Subsequent to 90Y-IT, the CR rate increased from 16 to 75% in the trial reported by Jurzak et al. [5], resulting in a median PFS and OS of 1.8 and 2.2 years, respectively. In a small trial with relapsed or refractory MCL patients with a median of 3 prior therapies, including high-dose chemotherapy (HCT) and autologous stem cell transplantation (ASCT), 90Y-IT was applied as a monotherapy in 16 patients and as consolidation after chemoimmunotherapy in 32 individuals. The overall response rate and CR for the entire patient cohort were 61 and 32%, respectively, and the median PFS and OS amounted to 6.2 and 25.5 months, respectively. When 90Y-IT was employed as consolidation, the ORR and CR rates were higher in comparison to 90Y-IT monotherapy, i.e. ORR 72 compared to 40% and CR 38 compared to 20%, respectively. The same was true for PFS and OS, i.e. PFS was 8.9 compared to 3.7 months and OS of 31.2 compared to 13.8 months [6]. In a single-centre trial with 90Y-IT monotherapy for patients with relapsed or refractory MCL and a median of 3 prior therapies, the ORR was 31%, CR was 15%, PFS was 6 months, and OS was 21 months. Interestingly, PFS in patients with either CR or PR amounted to 28 months. These data indicate that 90Y-IT may be more effective as consolidation after chemo(immune)therapy and has probably little effect if employed as monotherapy at relapse [12]. Unfortunately, there is no data available from the RIT-NT regarding the response rate prior to 90Y-IT consolidation to strengthen this notion, as response rates were exclusively reported after 90Y-IT.

In line with our data, PFS and OS for the whole group of relapsed or refractory patients amounted to 0.88 (10.8 months) and 3.85 years (46 months). In comparison, the Bruton’s kinase inhibitor ibrutinib alone induced a median PFS of 13.9 and 15.8 months after a median of three or two prior therapies and median follow-up of 15.3 months and 39 months, respectively, in two prospective clinical trials [13–15]. Overall survival amounted to 58% at 18 months and a median of 30.3 months in these two clinical studies, respectively. This demonstrates the efficacy of ibrutinib and underscores its role in relapsed or refractory MCL.

In conclusion, we report results from the largest registry of MCL pts. treated with 90Y-IT to date. Our data and results from prospective clinical trials demonstrate that 90Y-IT containing treatment of mantle cell lymphoma is feasible and achieves results comparable to chemoimmunotherapy. Data from clinical trials indicate that 90Y-IT may improve clinical results and may facilitate fewer cycles of chemoimmunotherapy. In our study, as a limitation of the registry data, response rates after induction chemoimmunotherapy, i.e. before 90Y-IT consolidation, were not reported. Therefore, we cannot tell whether 90Y-IT, when given as a consolidation after induction chemoimmunotherapy, improved the response rate achieved by induction treatment. To further elucidate this issue, a prospective clinical trial would be required. Of note, the results with 90Y-IT after chemoimmunotherapy at relapse are far less encouraging compared to treatment with small molecules such as ibrutinib.
